# Supplementary material for: A postsynaptic PI3K-cII dependent signaling controller for presynaptic homeostatic plasticity
Source: eLife. 2018 Jan 5;7:e31535. doi: 10.7554/eLife.31535 (PMC5773188; doi:10.7554/eLife.31535)
Supplement: Supplementary file 1. — Data includes mEPSP amplitudes, EPSP or EPSC amplitudes, quantal contents, and sample size (N). [file elife-31535-supp1.docx]

Supplementary File 1- Electrophysiological Data

|  | *Genotype* | PhTX  (+/-) | [Ca^2+^]_e_  ^­^(mM) | mEPSP Amplitude (mV) | EPSP Amplitude (mv) or EPSC Amplitude (nA) | Quantal Content | N |
| --- | --- | --- | --- | --- | --- | --- | --- |
| 1B  1C | *Sca-GAL4/+;BG57-GAL4/+* | - | 0.35 | 1.08 ± 0.03 | 32.6 ± 1.8 | 30.5 ± 1.9 | 20 |
|  | *Sca-GAL4/+;BG57-GAL4/+* | + | 0.35 | 0.51 ± 0.02 | 33.4 ± 1.2 | 67.7 ± 3.4 | 24 |
|  | *OK371-GAL4,BG57-GAL4/+* | - | 0.35 | 0.91 ± 0.03 | 35.9 ± 0.9 | 41.0 ± 1.4 | 43 |
|  | *OK371-GAL4,BG57-GAL4/+* | + | 0.35 | 0.47 ± 0.02 | 32.4 ± 0.7 | 75.2 ± 2.6 | 81 |
|  | *OK371-GAL4/+* | - | 0.35 | 0.70 ± 0.03 | 32.0 ± 1.2 | 46.1 ± 1.6 | 14 |
|  | *OK371-GAL4/+* | + | 0.35 | 0.34 ± 0.01 | 27.2 ± 0.9 | 83.2 ± 4.3 | 19 |
|  | *MHC-GAL4/+* | - | 0.35 | 0.96 ± 0.05 | 35.8 ± 1.2 | 39.3 ± 2.6 | 17 |
|  | *MHC-GAL4/+* | + | 0.35 | 0.36 ± 0.03 | 29.4 ± 1.1 | 86.4 ± 4.4 | 17 |
| 1D  1E | *Sca-GAL4/+; BG57-GAL4/ UAS-34621-RNAi* | - | 0.35 | 1.23 ± 0.07 | 33.6 ± 2.6 | 28.0 ± 2.6 | 9 |
|  | *Sca-GAL4/+; BG57-GAL4/ UAS-34621-RNAi* | + | 0.35 | 0.50 ± 0.09 | 17.2 ± 2.8 | 41.1 ± 2.3 | 11 |
| 2B  2C  2D | *w^1118^* | - | 0.3 | 0.81 ± 0.03 | 25.3 ± 1.4 | 31.4 ±1.9 | 15 |
|  | *w^1118^* | + | 0.3 | 0.34 ± 0.02 | 22.6 ± 1.7 | 52.6 ± 4.7 | 10 |
|  | *Pi3K68D^GS^* | - | 0.3 | 1.17 ± 0.04 | 31.2 ± 1.9 | 26.9 ± 2.2 | 6 |
|  | *Pi3K68D^GS^* | + | 0.3 | 0.45 ± 0.03 | 11.3 ± 1.7 | 26.02±3.8 | 9 |
|  | *Pi3K68D^MB^* | - | 0.3 | 0.80 ± 0.05 | 24.0 ± 2.7 | 30.8 ± 3.5 | 12 |
|  | *Pi3K68D^MB^* | + | 0.3 | 0.39 ± 0.03 | 13.8 ± 2.4 | 35.9 ± 5.3 | 11 |
|  | *Pi3K68D^AH1^* | - | 0.3 | 0.79 ± 0.06 | 20.3 ± 1.5 | 26.8 ± 2.4 | 14 |
|  | *Pi3K68D^AH1^* | + | 0.3 | 0.41 ± 0.02 | 11.3 ± 1.6 | 26.5 ± 2.8 | 14 |
| 3B | *w^1118^* | - | 0.3 | 0.91 ± 0.05 | 25.6 ± 1.4 | 28.7 ± 1.3 | 15 |
|  | *GluRIIA^sp16^* | - | 0.3 | 0.35 ± 0.02 | 17.2 ± 1.7 | 52.7 ± 6.2 | 18 |
|  | *Pi3K68D^AH1^* | - | 0.3 | 0.87 ± 0.04 | 18.2 ± 1.2 | 21.3 ± 1.8 | 16 |
|  | *GluRIIA^sp16^; Pi3K68D^AH1^* | - | 0.3 | 0.29 ± 0.01 | 7.4 ± 1.0 | 25.7 ± 3.8 | 19 |
| 4A | *w^1118^* | - | 0.3 | 0.87 ± 0.06 | 19.8 ± 2.0 | 22.5 ± 2.0 | 10 |
|  | *w^1118^* | + | 0.3 | 0.40 ± 0.03 | 21.7 ± 2.4 | 55.4 ± 5.2 | 10 |
|  | *OK371-GAL4/UAS-Pi3K68D-GFP* | - | 0.3 | 0.79 ± 0.03 | 24.0 ± 2.6 | 30.0 ± 2.7 | 13 |
|  | *OK371-GAL4/UAS-Pi3K68D-GFP* | + | 0.3 | 0.39 ± 0.02 | 25.0 ± 2.1 | 64.9 ± 5.7 | 16 |
|  | *Pi3K68D^AH1^* | - | 0.3 | 0.85 ± 0.06 | 18.7 ± 2.6 | 21.7 ± 3.7 | 12 |
|  | *Pi3K68D^AH1^* | + | 0.3 | 0.42 ± 0.04 | 7.0 ± 1.8 | 17.3 ± 3.9 | 9 |
|  | *OK371-GAL4/UAS-Pi3K68D-GFP; Pi3K68D^AH1^* | - | 0.3 | 0.70 ± 0.04 | 13.8 ± 1.6 | 20.0 ± 2.6 | 13 |
|  | *OK371-GAL4/UAS-Pi3K68D-GFP; Pi3K68D^AH1^* | + | 0.3 | 0.32 ± 0.02 | 4.5 ± 0.8 | 14.3 ± 2.1 | 15 |
| 4B | *UAS-Pi3K68D-GFP/+* | - | 0.3 | 0.83 ± .06 | 30.7 ± 1.7 | 39.1 ± 3.2 | 11 |
|  | *UAS-Pi3K68D-GFP/+* | + | 0.3 | 0.30 ± 0.03 | 21.9 ± 1.7 | 74.3 ± 5.0 | 11 |
|  | *Pi3K68D^AH1^* | - | 0.3 | 0.83 ± 0.05 | 23.8 ± 1.8 | 29.5 ± 2.6 | 15 |
|  | *Pi3K68D^AH1^* | + | 0.3 | 0.34 ± 0.02 | 13.3 ± 1.7 | 39.3 ± 4.5 | 9 |
|  | *UAS-Pi3K68D-GFP/+; BG57-GAL4, Pi3K68D^AH1^/ Pi3K68D^AH1^* | - | 0.3 | 0.58 ± 0.04 | 17.7 ± 1.3 | 29.6 ± 2.4 | 19 |
|  | *UAS-Pi3K68D-GFP/+; BG57-GAL4, Pi3K68D^AH1^/ Pi3K68D^AH1^* | + | 0.3 | 0.28 ± 0.01 | 20.4 ± 1.5 | 63.3 ± 6.9 | 11 |
| 4E | *BG57-GAL4/+* | - | 0.3 | 0.88 ± 0.06 | 31.6 ± 1.7 | 36.9 ± 2.5 | 10 |
|  | *BG57-GAL4/+* | + | 0.3 | 0.43 ± 0.02 | 27.2 ± 2.1 | 64.4 ± 5.0 | 12 |
|  | *UAS-mcherry-Pi3K68D^kinase-dead ∆21^/+* | - | 0.3 | 0.98 ± 0.05 | 21.0 ± 1.8 | 22.0 ± 1.8 | 10 |
|  | *UAS-mcherry-Pi3K68D^kinase-dead ∆21^/+* | + | 0.3 | 0.43 ± 0.03 | 18.3 ± 1.8 | 43.8 ± 34.8 | 10 |
|  | *UAS-mcherry-Pi3K68D^kinase-dead ∆21^/+; BG57-GAL4/+* | - | 0.3 | 0.84 ± 0.07 | 30.1 ± 2.5 | 36.6 ± 3.0 | 14 |
|  | *UAS-mcherry-Pi3K68D^kinase-dead ∆21^/+; BG57-GAL4/+* | + | 0.3 | 0.39 ± 0.02 | 17.8 ± 1.7 | 47.2 ± 4.5 | 12 |
|  | *UAS-mcherry-Pi3K68D^∆N^/+; BG57-GAL4/+* | - | 0.3 | 0.93 ± 0.08 | 23.3 ± 2.5 | 26.12 ± 2.6 | 11 |
|  | *UAS-mcherry-Pi3K68D^∆N^/+; BG57-GAL4/+* | + | 0.3 | 0.38 ± 0.02 | 13.6 ± 3.0 | 34.4 ± 6.9 | 11 |
| 5B | *Pi3K68D^AH1^/+* | - | 0.3 | 0.64 ± 0.03 | 15.7 ± 1.8 | 25.1 ± 3.2 | 11 |
|  | *Pi3K68D^AH1^/+* | + | 0.3 | 0.36 ± 0.04 | 21.0 ± 1.9 | 65.4 ± 8.3 | 15 |
|  | *rim^103^/+* | - | 0.3 | 0.95 ± 0.11 | 19.0 ± 3.4 | 21.3 ± 5.2 | 6 |
|  | *rim^103^/+* | + | 0.3 | 0.35 ± 0.02 | 16.8 ± 2.2 | 48.9 ± 7.6 | 8 |
|  | *dmp^f0^/+* | - | 0.3 | 0.84 ± 0.06 | 23.1 ± 1.9 | 28.9 ± 3.5 | 7 |
|  | *dmp ^f0^/+* | + | 0.3 | 0.38 ± 0.04 | 23.9 ± 1.4 | 65.4 ± 7.1 | 7 |
|  | *Pi3K68D^AH1^/ rim^103^* | - | 0.3 | 0.73 ± 0.05 | 16.8 ± 1.7 | 24.6 ± 3.0 | 9 |
|  | *Pi3K68D^AH1^/ rim^103^* | + | 0.3 | 0.33 ± 0.01 | 5.8 ± 0.8 | 17.9 ± 2.6 | 11 |
|  | *Pi3K68D^AH1^/ dmp^f0^* | - | 0.3 | 0.71 ± 0.03 | 20.4 ± 2.0 | 28.4 ± 2.5 | 12 |
|  | *Pi3K68D^AH1^/ dmp^f0^* | + | 0.3 | 0.31 ± 0.02 | 11.9 ± 1.5 | 38.8 ± 3.9 | 12 |
| 5-S1 | *BG57-GAL4/+* | - | 0.3 | 0.86 ± 0.1 | 29.2 ± 2.2 | 35.1 ± 3.2 | 6 |
|  | *BG57-GAL4/+* | + | 0.3 | 0.47 ± 0.02 | 29.2 ± 3.0 | 63.6 ± 7.2 | 6 |
|  | *BG57-GAL4, Pi3K68D^AH1^/ Pi3K68D^AH1^* | - | 0.3 | 0.87 ± 0.09 | 21.6 ± 1.6 | 25.4 ± 1.8 | 6 |
|  | *BG57-GAL4, Pi3K68D^AH1^/ Pi3K68D^AH1^* | + | 0.3 | 0.30 ± 0.02 | 8.0 ± 1.2 | 26.1 ± 3.4 | 6 |
|  | *UAS-endostatin/+; BG57-GAL4, Pi3K68D^AH1^/ Pi3K68D^AH1^* | - | 0.3 | 0.57 ± 0.05 | 12.7 ± 0.9 | 24.2 ± 2.7 | 12 |
|  | *UAS-endostatin/+; BG57-GAL4, Pi3K68D^AH1^/ Pi3K68D^AH1^* | + | 0.3 | 0.36 ± 0.04 | 8.9 ± 1.5 | 24.1 ± 2.5 | 8 |
| 7A | *BG57-GAL4/+* | - | 0.3 | 1.05 ± 0.1 | 32.4 ± 3.2 | 6 | 6 |
|  | *BG57-GAL4/+* | + | 0.3 | 0.44 ± 0.05 | 28.7 ± 2.4 | 6 | 6 |
|  | *uas-rab11 RNAi / BG57-GAL4/+* | - | 0.3 | 0.84 ± 0.05 | 27.9 ± 2.9 | 12 | 8 |
|  | *uas-rab11 RNAi / BG57-GAL4/+* | + | 0.3 | 0.45 ± 0.02 | 18.6 ± 2.1 | 8 | 10 |
| 7B | *UAS-Pi3K92E RNAi/ MHC-GAL4* | - | 0.35 | 0.72 ± 0.06 | 32.5 ± 1.7 | 46.7 ± 4.9 | 5 |
|  | *UAS-Pi3K92E RNAi / MHC-GAL4* | + | 0.35 | 0.36 ± 0.01 | 29.0 ± 1.5 | 79.7 ± 4.3 | 7 |
|  | *GMR-GAL4/+; BG57-GAL4/uas-PTEN* | - | 0.3 | 0.68 ± 0.04 | 18.97 ± 1.8 | 26.5 ± 2.3 | 8 |
|  | *GMR-GAL4/+; BG57-GAL4/uas-PTEN* | + | 0.3 | 0.36 ± 0.02 | 20.4 ± 2.0 | 57.1 ± 4.73 | 7 |
| 7C | *UAS-Vps34 RNAi/ MHC-GAL4* | - | 0.35 | 0.79 ± 0.04 | 38.8 ± 1.8 | 50.2 ± 4.3 | 8 |
|  | *UAS-Vps34 RNAi/MHC-GAL4* | + | 0.35 | 0.44 ± 0.04 | 25.9 ± 2.3 | 62.0 ± 6.7 | 11 |
|  | *w^1118^* | - | 0.5 | 0.62 ± 0.03 | 84.5 ± 9.5 | 140.5 ± 17.2 | 14 |
|  | *w^1118^* | + | 0.5 | 0.32 ± 0.009 | 68.8 ± 5.8 | 218.5 ± 21.8 | 12 |
|  | *Pi3K68D^AH1^* | - | 0.5 | 0.67 ± 0.05 | 63.8 ± 6.7 | 103.6 ± 16.0 | 10 |
|  | *Pi3K68D^AH1^* | + | 0.5 | 0.31 ± 0.02 | 30.3 ± 3.7 | 101.7 ± 13.7 | 10 |
| 8B  8D | *w^1118^* | - | 0.7 | 0.63 ± 0.04 | 173.3 ± 17.1 | 283.0 ± 39.7 | 6 |
|  | *w^1118^* | + | 0.7 | 0.34 ± 0.06 | 119.6 ± 19.4 | 406.0 ± 94.4 | 6 |
|  | *Pi3K68D^AH1^* | - | 0.7 | 0.75 ± 0.06 | 100.6 ± 14.1 | 133.1 ± 17.4 | 7 |
|  | *Pi3K68D^AH1^* | + | 0.7 | 0.26 ± 0.01 | 53.9 ± 3.93 | 211.4 ± 24.8 | 8 |
|  | *w^1118^* | - | 1.0 | 0.72 ± 0.04 | 234.7 ± 16.1 | 333.2 ± 32.4 | 10 |
|  | *w^1118^* | + | 1.0 | 0.35 ± 0.02 | 197.2 ± 18.2 | 605.4 ± 77.5 | 11 |
|  | *Pi3K68D^AH1^* | - | 1.0 | 0.73 ± 0.03 | 185.7 ± 10.9 | 261.0 ± 19.0 | 11 |
|  | *Pi3K68D^AH1^* | + | 1.0 | 0.40 ± 0.01 | 141.1 ± 8.8 | 363.6 ± 27.0 | 14 |
|  | *w^1118^* | - | 1.5 | 0.64 ± 0.04 | 280.5 ± 13.5 | 465.4 ± 32.7 | 13 |
|  | *w^1118^* | + | 1.5 | 0.32 ± 0.01 | 229.7 ± 11.7 | 745.3 ± 62.6 | 15 |
|  | *Pi3K68D^AH1^* | - | 1.5 | 0.69 ± 0.04 | 221.7 ± 12.0 | 340.1 ± 26.6 | 19 |
|  | *Pi3K68D^AH1^* | + | 1.5 | 0.33 ± 0.01 | 174.2 ± 8.18 | 534.2 ± 28.6 | 9 |
|  | *Pi3K68D^AH1^* | - | 1.5 | 0.63 ± 0.04 | 250.1 ± 11.8 | 416.8 ± 36.3 | 7 |
|  | *GluRIIA^sp16^; Pi3K68D^AH1^* | - | 1.5 | 0.25 ± 0.02 | 176.2 ± 10.1 | 775.4 ± 56.7 | 6 |
|  | *UAS-mcherry-Pi3K68D^kinase-dead ∆21^/+* | - | 1.5 | 0.57 ± 0.04 | 229.2 ± 17.1 | 414.2 ± 32.2 | 10 |
|  | *UAS-mcherry-Pi3K68D^kinase-dead ∆21^/+* | + | 1.5 | 0.26 ± 0.02 | 217.3 ± 20.0 | 828.6 ± 40.1 | 10 |
| 8C | *UAS-mcherry-Pi3K68D^kinase-dead ∆21^/+; BG57-GAL4/+* | - | 1.5 | 0.53 ± 0.07 | 232.9 ± 23.9 | 430.1 ± 35.7 | 14 |
|  | *UAS-mcherry-Pi3K68D^kinase-dead ∆21^/+; BG57-GAL4/+* | + | 1.5 | 0.30 ± 0.01 | 222.6 ± 23.0 | 749.9 ± 97.1 | 12 |
| 8 | *BG57-GAL4/+* | - | 1.5 | 0.71 ± 0.09 | 196.3 ± 10.3 | 308.8 ± 32.5 | 10 |
|  | *BG57-GAL4/+* | + | 1.5 | 0.34 ± 0.03 | 174.8 ± 16.6 | 573.6 ± 80.9 | 10 |
|  | *BG57-GAL4/uas-Rab11-RNAi* | - | 1.5 | 0.54 ± 0.03 | 186.4 ± 9.3 | 359.7 ± 32.5 | 10 |
|  | *BG57-GAL4/uas-Rab11-RNAi* | + | 1.5 | 0.40 ± 0.02 | 209.8 ± 9.6 | 531.6 ± 26.4 | 11 |
|  | *BG57-GAL4/uas-Vps34-RNAi* | - | 1.5 | 0.67 ± 0.04 | 209.4 ± 12.7 | 318.7 ± 18.9 | 12 |
|  | *BG57-GAL4/uas-Vps34-RNAi* | + | 1.5 | 0.33 ± 0.02 | 165.7 ± 9.8 | 508.3 ± 33.6 | 9 |
| 8-S1 | *w^1118^* | - | 1.0 | 0.72 ± 0.04 | 234.7 ± 16.1 | 333.2 ± 32.4 | 10 |
|  | *w^1118^* | + | 1.0 | 0.35 ± 0.02 | 197.2 ± 18.2 | 605.4 ± 77.5 | 11 |
|  | *Pi3K68D^AH1^* | - | 1.0 | 0.73 ± 0.03 | 185.7 ± 10.9 | 261.0 ± 19.0 | 11 |
|  | *Pi3K68D^AH1^* | + | 1.0 | 0.40 ± 0.01 | 141.1 ± 8.8 | 363.6 ± 27.0 | 14 |
|  | *Vps34^m22^/+; Pi3K68D^AH1^* | - | 1.0 | 0.67 ± 0.05 | 140.9 ± 11.2 | 218.9 ± 22.4 | 10 |
|  | *Vps34^m22^/+; Pi3K68D^AH1^* | + | 1.0 | 0.36 ± 0.19 | 129.3 ± 11.0 | 357.5 ± 25.2 | 10 |

|  | Genotype | PhTX  (+/-) | [Ca^2+^]_e_ | mEPSC (mV) | Cum. EPSC (nA) | RRP | Ptrain | EPSC4/  EPSC1 | N |
| --- | --- | --- | --- | --- | --- | --- | --- | --- | --- |
| 8F | *w^1118^* | - | 1.5 | 0.59 ± 0.06 | 1125 ± 57.5 | 2026 ± 168.2 | 0.30 ± 0.008 | 0.68 ± 0.02 | 10 |
| 8F | *Pi3K68D^AH1^* | - | 1.5 | 0.70 ± 0.09 | 1020 ± 65.77 | 1563 ± 179.2 | 0.26 ± 0.01 | 0.83 ± 0.03 | 7 |
