# Supplementary material for: A postsynaptic PI3K-cII dependent signaling controller for presynaptic homeostatic plasticity
Source: eLife. 2018 Jan 5;7:e31535. doi: 10.7554/eLife.31535 (PMC5773188; doi:10.7554/eLife.31535)
Supplement: Transparent reporting form [file elife-31535-transrepform.docx]

***eLife’s* transparent reporting form**

We encourage authors to provide detailed information *within their submission* to facilitate the interpretation and replication of experiments. Authors can upload supporting documentation to indicate the use of appropriate reporting guidelines for health-related research (see [EQUATOR Network](http://www.equator-network.org/%20)), life science research (see the [BioSharing Information Resource](https://biosharing.org/)), or the [ARRIVE guidelines](http://www.plosbiology.org/article/info:doi/10.1371/journal.pbio.1000412) for reporting work involving animal research. Where applicable, authors should refer to any relevant reporting standards documents in this form.

If you have any questions, please consult our Journal Policies and/or contact us: [editorial@elifesciences.org](mailto:editorial@elifesciences.org).

**Sample-size estimation**

- You should state whether an appropriate sample size was computed when the study was being designed
- You should state the statistical method of sample size computation and any required assumptions
- If no explicit power analysis was used, you should describe how you decided what sample (replicate) size (number) to use

A power analysis was performed for our experiments. Our effect size is generally quite large, 75-150%, with low variance. As a consequence, sample sizes of 5 or more independent recordings were considered adequately powered. Generally, our samples sizes vastly exceed this number. Throughout our study, control genotypes, whether wild type or genotypic controls, are repeated at the same time that the experimental genotypes are tested. Thus, over the course of the four years it took to complete this study, controls were often repeated many times. Sample sizes can be found in the supplementary data file 1 for electrophysiology experiments and in the figure legends for imaging experiments.

A Student’s t-test is used to compare a single genotype in the presence and absence of toxin application. ANOVA was used for multiple comparisons, as indicated in the text and methods.

Please outline where this information can be found within the submission (e.g., sections or figure legends), or explain why this information doesn’t apply to your submission:

**Replicates**

- You should report how often each experiment was performed
- You should include a definition of biological versus technical replication
- The data obtained should be provided and sufficient information should be provided to indicate the number of independent biological and/or technical replicates
- If you encountered any outliers, you should describe how these were handled
- Criteria for exclusion/inclusion of data should be clearly stated
- High-throughput sequence data should be uploaded before submission, with a private link for reviewers provided (these are available from both GEO and ArrayExpress)

Please outline where this information can be found within the submission (e.g., sections or figure legends), or explain why this information doesn’t apply to your submission:

All data sets that are presented for a given genotype in our manuscript represent intracellular recordings from at least two independent genetic crosses (biological replicate) and at least two animals per cross (another form of biological replicate) and numerous intracellular recordings from independent NMJ (technical replicates). This is stated in our methods section.

We provide a data table, inclusive of more than 1200 intracellular electrophysiology recordings, that details the numerical values associated with the data in our figures. Exact sample sizes are given as are values for each of the underlying measures. Each data set is referenced to the figure in which the data reside.

We have used standard criteria for ascertaining whether an intracellular recording electrode has damaged the muscle cell during impalement, based on cellular input resistance and resting membrane potential. These criteria are independent of the measures that are used in our analyses and have been routinely employed in our publications, and that of others in the field, for the past 10 years. These criteria are stated in the methods section under ‘electrophysiology’. Similarly, a threshold decrease in mEPSP amplitude, below average baseline, was used to confirm the activity of PhTX, as described in the methods section under ‘electrophysiology.’

All data that meet our criteria for healthy, stable recordings are used. No outliers are identified or removed from our analyses. For imaging/ anatomical analyses, no muscles were excluded from analysis unless visibly damaged during dissection.

**Statistical reporting**

- Statistical analysis methods should be described and justified
- Raw data should be presented in figures whenever informative to do so (typically when N per group is less than 10)
- For each experiment, you should identify the statistical tests used, exact values of N, definitions of center, methods of multiple test correction, and dispersion and precision measures (e.g., mean, median, SD, SEM, confidence intervals; and, for the major substantive results, a measure of effect size (e.g., Pearson's r, Cohen's d)
- Report exact p-values wherever possible alongside the summary statistics and 95% confidence intervals. These should be reported for all key questions and not only when the p-value is less than 0.05.

Please outline where this information can be found within the submission (e.g., sections or figure legends), or explain why this information doesn’t apply to your submission:

Statistical methods are reported in the manuscript, figure legends and methods section.

An extensive data table presents all data underlying the recordings in our figures. This includes data from more than 1200 recordings. Each data set included in this data table is referenced to the figure in which the data are presented.

Data from our genetic screens are presented as individual data points.

Statistical results are presented in figures and figure legends.

(For large datasets, or papers with a very large number of statistical tests, you may upload a single table file with tests, Ns, etc., with reference to sections in the manuscript.)

**Group allocation**

- Indicate how samples were allocated into experimental groups (in the case of clinical studies, please specify allocation to treatment method); if randomization was used, please also state if restricted randomization was applied
- Indicate if masking was used during group allocation, data collection and/or data analysis

Please outline where this information can be found within the submission (e.g., sections or figure legends), or explain why this information doesn’t apply to your submission:

Allocation was based on animal genotype. For *Drosophila* genetics, this is straightforward.

Masking was not used during genotype allocation. Foundational results were independently replicated by two investigators and routinely re-assessed, over time. Foundational mutant phenotypes were also independently replicated in multiple conditions and results are reported at these independent conditions.

**Additional data files (“source data”)**

- We encourage you to upload relevant additional data files, such as numerical data that are represented as a graph in a figure, or as a summary table
- Where provided, these should be in the most useful format, and they can be uploaded as “Source data” files linked to a main figure or table
- Include model definition files including the full list of parameters used
- Include code used for data analysis (e.g., R, MatLab)
- Avoid stating that data files are “available upon request”

Please indicate the figures or tables for which source data files have been provided:

An extensive data table presents all data underlying the recordings in our figures. This includes data from more than 1200 recordings. Each data set included in this data table is referenced to the figure in which the data are presented. Code for data analysis is previously published.
